# Master regulators of skeletal muscle lineage development and pluripotent stem cells differentiation

**DOI:** 10.1186/s13619-021-00093-5

**Published:** 2021-10-01

**Authors:** Joana Esteves de Lima, Frédéric Relaix

**Affiliations:** Univ Paris Est Creteil, INSERM, EnvA, EFS, AP-HP, IMRB, 94010 Creteil, France

**Keywords:** Myogenesis, Muscle progenitor, PAX3, PAX7, MRF, MYF5, MYOD, MYOG, hiPSC, ESC

## Abstract

In vertebrates, the skeletal muscles of the body and their associated stem cells originate from muscle progenitor cells, during development. The specification of the muscles of the trunk, head and limbs, relies on the activity of distinct genetic hierarchies. The major regulators of trunk and limb muscle specification are the paired-homeobox transcription factors PAX3 and PAX7. Distinct gene regulatory networks drive the formation of the different muscles of the head. Despite the redeployment of diverse upstream regulators of muscle progenitor differentiation, the commitment towards the myogenic fate requires the expression of the early myogenic regulatory factors MYF5, MRF4, MYOD and the late differentiation marker MYOG. The expression of these genes is activated by muscle progenitors throughout development, in several waves of myogenic differentiation, constituting the embryonic, fetal and postnatal phases of muscle growth. In order to achieve myogenic cell commitment while maintaining an undifferentiated pool of muscle progenitors, several signaling pathways regulate the switch between proliferation and differentiation of myoblasts. The identification of the gene regulatory networks operating during myogenesis is crucial for the development of in vitro protocols to differentiate pluripotent stem cells into myoblasts required for regenerative medicine.

## Background

Skeletal muscles constitute the most abundant tissue in the vertebrate body. The voluntary contraction of the muscles requires electrical stimuli provided by motoneurons to the fibers. Then, the mechanical forces generated by muscle fiber contraction are transmitted to the bones (or connective tissues) by the tendons. The contractibility of the fibers relies on the structural organization of the actin and myosin proteins that constitute the sarcomeres.

**Fig. 1:**
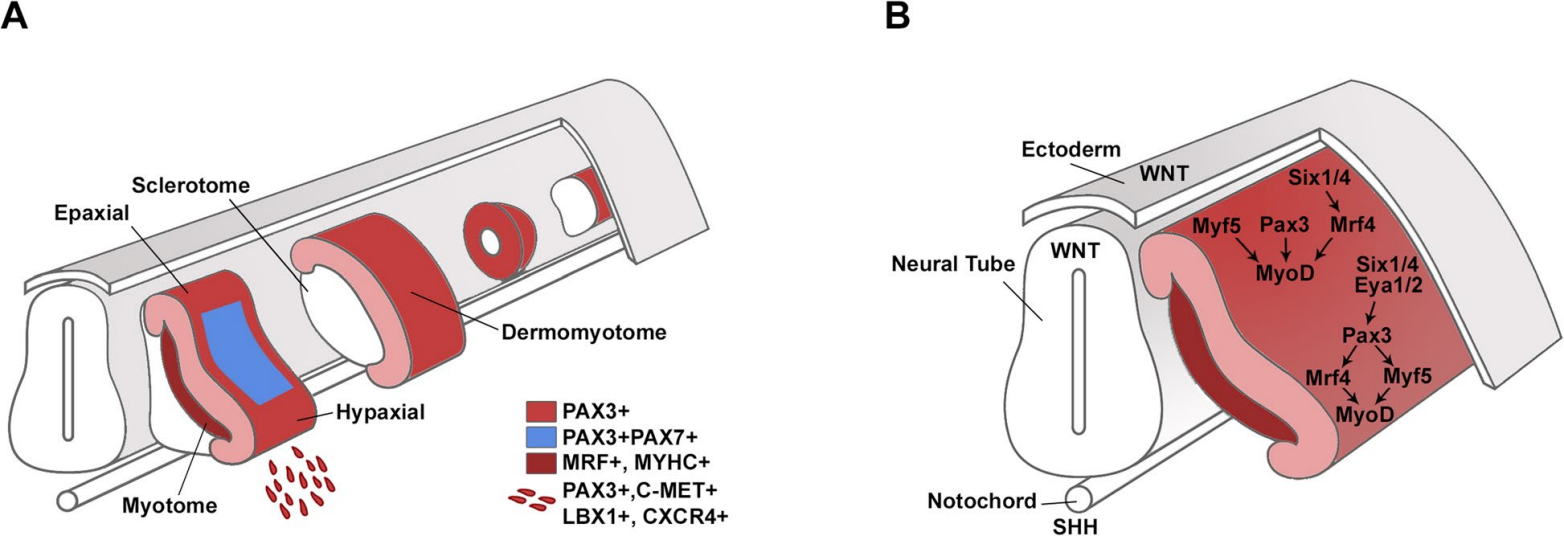
Dermomyotome development and skeletal muscle determination. **A** Scheme depicting the different degrees of somite maturation, with the dermomyotomal domains highlighted. **B** Signaling cascades operating at the epaxial and hypaxial dermomyotomal domains, which activate the expression of the MRFs

In tetrapods, skeletal muscles from the trunk and limbs derive from the somites, while head muscles originate from the cranial and prechordal mesoderm. During development, paraxial mesoderm in the trunk undergoes segmentation, forming the somites, which are transient mesodermal structures that subsequently mature into distinct layers, the dermomyotome, the myotome, the syndetome and the sclerotome (Fig. 1A) (Buckingham and Rigby, 2014). Dorsally, the dermomyotome gives rise to the muscles of the trunk and limbs, the dermis of the back, and contributes to the formation of vascular and brown adipocyte tissue cells. Ventrally, the syndetome and the sclerotome form the tendons and bones of the trunk, respectively. The cells from the epaxial (medial) and hypaxial (lateral) edges of the dermomyotome undergo epithelial-to-mesenchymal transition (EMT) and delaminate, migrate ventrally and give rise to the myotome, where the first myoblast fusion events take place (Fig. 1A) (Buckingham and Relaix, 2007). In a second wave of colonization of the myotome, the progenitor cells present in the central dermomyotome delaminate and migrate ventrally, intercalating within the primary fibers of the myotome. Within the myotome, these cells can either differentiate into myoblasts or self-renew, maintaining an undifferentiated progenitor pool. Subsequently, the myotome expands ventrally, and complex patterning events will give rise to trunk muscles. Laterally, the delaminating cells migrate to distant locations and provide the muscles of the limbs, diaphragm and the hypoglossal cord (Buckingham and Rigby, 2014). The skeletal muscles of the head derive mostly from the cranial mesoderm, while the extraocular muscles originate from the most anterior mesoderm, the prechordal mesoderm (Comai et al., 2020; Noden and Francis-West, 2006). The formation of most of the head muscles relies on the branchial arches which, like the somites, are transient mesodermal structures that align ventrally, along the pharynx. Although the master regulators of skeletal muscle commitment are shared between head and trunk muscles, muscle specification in these regions relies on distinct upstream transcription factors.

## Main Text

### PAX transcription factors and myogenic specification

Skeletal muscle development of the trunk and limbs relies on the presence of progenitor cells that express the paired-homeobox transcription factors PAX3 and PAX7. These genes are major upstream regulators of myogenesis but their expression is not muscle-specific, being also present in neural crest cells and neuro-ectoderm, in the dorsal part of the neural tube and in the central nervous system, where they play essential roles in tissue specification and organ development (Buckingham and Relaix, 2007). PAX3 is expressed in the paraxial mesoderm and in the early epithelial somite but its expression becomes restricted to the dermomyotome in the mature somite. The expression of PAX7 however, is induced later in the central dermomyotome, where it co-localizes with PAX3. Studies performed in chick and mouse embryos identified this group of cells co-expressing PAX3 and PAX7 and demonstrated that it contributes to the formation of all the muscles of the trunk and limbs and to the associated muscle stem cells (satellite cells) (Gros et al., 2005; Kassar-Duchossoy, 2005; Relaix et al., 2005; Schienda et al., 2006). PAX3 and PAX7 share conserved DNA binding domains, and display partial functional overlap in myogenesis (Relaix et al., 2004, 2006). In *Pax3*-deficient embryos, trunk muscle formation is impaired, cells of the hypaxial domain of the dermomyotome undergo cell death and consequently limb muscles and other muscles of migratory origin are absent (Table 1) (Bober et al., 1994; Epstein et al., 1996; Goulding et al., 1994; Relaix et al., 2003; Tremblay et al., 1998). The severe phenotype associated with the hypaxial dermomyotome in *Pax3*-mutant embryos is consistent with higher PAX3 expression in the hypaxial domain (Kassar-Duchossoy, 2005; Relaix et al., 2005). In contrast, deleting PAX7 does not affect embryonic development (Table 1) (Mansouri et al., 1996; Relaix et al., 2006; Seale et al., 2000). However, *Pax7*-deficient mice progressively lose the satellite cell pool, which compromises muscle homeostasis and regeneration (Table 1). This is consistent with PAX7 being expressed in all adult muscle stem cells and required for their self-renewal, survival, propagation and maintenance (Addicks et al., 2019; Kuang et al., 2006; Lepper et al., 2009; Oustanina et al., 2004; Relaix et al., 2006; Seale et al., 2000; Sincennes et al., 2021). Furthermore, mouse embryos lacking PAX3 and PAX7 display a complete loss of trunk and limb muscles with only the primary myotome being formed (Table 1) (Relaix et al., 2005). Therefore, PAX3 and PAX7 cannot compensate for one another during development or in satellite cell maintenance, which could be associated with the presence of distinct downstream gene regulatory networks.

**Table 1:** List of the phenotypes associated with PAX3, PAX7 and MRF mutations

Mutated genes	Phenotype	Lethality	References
Pax3	Disorganized myotome Impaired trunk myogenesis No migratory muscles formed	Embryonic lethal (E15.5-E18.5	(Bober et al., 1994) (Goulding et al., 1994) (Epstein et al., 1996) (Tremblay et al., 1998) (Relaix et al., 2003
Pax7	Normal embryogenesis Reduced muscle masses Satellite cell loss after birth	Postnatal lethal (2 to 3 weeks after birth	(Mansouri et al., 1996) (Seale et al., 2000) (Oustanina et al., 2004) (Kuang et al., 2006) (Relaix et al., 2006)
Pax3:Pax7	No trunk muscles No muscles of migratory origin	Embryonic lethal	(Relaix et al., 2005)
Pax3:Myf5	No trunk muscles No muscles of migratory origin	Embryonic lethal	(Tajbakhsh et al., 1997)
Myf5	Delayed myotome formation Delayed epaxial development Normal musculature	Postnatal lethal (abnormal rib formation)	(Braun et al., 1992) (Braun et al., 1994) (Kablar et al., 1997) (Tajbakhsh et al., 1998)
Mrf4	Upregulation of Myog expression Normal musculature	Postnatal lethal (abnormal rib formation)	(Zhang et al., 1995) (Patapoutian et al., 1995)
Myod	Upregulation of Myf5 expression Delayed hypaxial development Normal musculature	Viable and fertile	(Rudnicki et al., 1992) (Kablar et al., 1997)
Myog	No myoblast differentiation Severely reduced musculature	Postnatal lethal	(Hasty et al., 1993) (Nabeshima et al., 1993)
Myod:Myf5:(Mrf4)	No embryonic myogenesis Lack of trunk and limb muscles	Postnatal lethal	(Rudnicki et al., 1993)
Myf5:Myod	MRF4-dependent embryonic myogenesis only Defective fetal myogenesis Lack of trunk and limb muscles	Postnatal lethal	(Kassar-Duchossoy et al., 2004)

Until recently, very few PAX3/PAX7 targets have been identified (Bajard et al., 2006; Epstein et al., 1996; Esteves de Lima et al., 2021a; Lagha et al., 2008; Sato et al., 2010). A chromatin immunoprecipitation followed by sequencing (ChIP-seq) performed in myoblasts ectopically expressing a tagged version of PAX3 and PAX7 identified a large set of target genes (Soleimani et al., 2012). Moreover, this analysis highlighted that in adult myoblasts, PAX7 shares similar binding sites as those of PAX3 but also recognizes newly identified regions in the loci of myogenic genes (Soleimani et al., 2012). In addition, while PAX3 displays more affinity for paired-box motifs on DNA, PAX7 mainly binds homeobox sites, which may contribute to the functional differences observed for each of these genes (Soleimani et al., 2012). A specific antibody recognizing the fusion protein PAX3-FOXO1 was used to identify PAX3-FOXO1 direct target genes (Cao et al., 2010). The PAX3-FOXO1 fusion protein contains the PAX3 DNA binding domains and the FOXO1 transactivation domain contributing to the majority of rhabdomyosarcoma cases via impairment of myogenic differentiation (Calhabeu et al., 2013). The vast majority of PAX3-FOXO1 binding sites were identified in introns and in distal intergenic regions conserved in vertebrates (Cao et al., 2010). In addition, a binding motif analysis revealed that there is a selective enrichment of PAX3-FOXO1 in genomic regions containing PAX3 and E-box motifs (Cao et al., 2010). More recently, PAX3 genomic occupancy was investigated in mouse and human embryonic stem cells (ESCs) ectopically expressing PAX3 (Magli et al., 2019a). The gene ontology terms associated with PAX3 detected peaks related to mesoderm regulation and skeletal muscle development (Magli et al., 2019a). Similar analyses were performed in mouse pluripotent stem cells (PSCs) ectopically expressing PAX7 (Lilja et al., 2017). PAX7 occupancy in these cells was associated with genes that regulate transcription, proliferation, muscle development, cell adhesion and migration (Lilja et al., 2017).

While the muscles of the trunk and limbs rely on PAX3 and PAX7 for skeletal muscle lineage specification, *Pax3* and *Pax7* are not expressed in the developing head muscles and in the *Pax3:Myf5* double mutant embryos the head muscles are spared (Tajbakhsh et al., 1997). The specification of skeletal muscle in the head relies on distinct genetic hierarchies, including the transcription factors Capsulin and MYOR, the T-box transcription factor 1 (TBX1) and the paired-like homeodomain transcription factor 2 (PITX2), that act genetically upstream of *Myf5*, *Mrf4* and *Myod* expression in separate head muscle groups. Mutations in any of these genes leads to distinct head muscle defects (Dong et al., 2006; Kelly et al., 2004; Lu et al., 2002, 1999; Noden and Francis-West, 2006).

### Master regulators of skeletal muscle determination

Distinct gene regulatory networks operate in different body regions (head, trunk, limbs) to activate the expression of the Myogenic Regulatory Factors (MRF) in muscle progenitors. The MRFs are a family of basic-Helix-Loop-Helix (bHLH) transcription factors that recognize the E-box consensus motif CANNTG in DNA. In order to bind DNA, MRF proteins need to heterodimerize with the ubiquitous bHLH E-proteins (Lassar et al., 1991). Expression of the MRF family of transcription factors is sufficient to drive a myogenic fate and the family includes MYF5, MYOD, MRF4 and MYOG. The first identified MRF was MYOD, which was able to convert fibroblasts into myoblasts, a landmark discovery for the understanding of myogenic regulation by MYOD and the first example of cell lineage identity reprogramming (Davis et al., 1987; Weintraub et al., 1991). While any of the MRFs can commit a non-myoblast cell to the myogenic lineage, loss of function experiments performed in mice have shown that MYF5, MYOD and MRF4 are associated with early myogenic determination while MYOG is a terminal differentiation driver of myogenesis (Hasty et al., 1993; Nabeshima et al., 1993). Different MRFs contain similar and conserved protein domains and show some degree of functional overlap. The constitutive ablation of *Myod* in mouse embryos does not lead to major muscle defects and *Myf5* expression is maintained and/or upregulated, indicating a compensatory mechanism (Table 1) (Kablar et al., 1997; Rudnicki et al., 1992). Conversely, in *Myf5*-null embryos skeletal muscle develops normally despite delayed *Myod* expression and primary myotome and epaxial-derived muscle formation (Table 1) (Braun et al., 1992, 1994; Kablar et al., 1997). The delayed expression of *Myod* in *Myf5* mutants indicate that MYOD have a more specific role in governing myogenesis epaxially. Analysis of the *Pax3;Myf5* double mutant embryos placed PAX3 and MYF5 hierarchically upstream of MYOD, since in these compound mutant embryos no trunk muscles are formed (Table 1) (Tajbakhsh et al., 1997). However, it remains unclear if this phenotype is linked to direct genetic interactions and/or combined specific cellular defects of *Pax3*- (loss of hypaxial and impaired epaxial dermomyotome) and *Myf5*-mutants (delayed myotome formation with progenitor cells stalled at the edges of the dermomyotome). Regarding MRFs compensation, combined ablations usually lead to severe skeletal muscle development impairment (Table 1), such as in the double knockout *Myf5;Myod* mutant (Kassar-Duchossoy et al., 2004) or when *Myod;Myf5;(Mrf4)* are deleted (Rudnicki et al., 1993). Conversely, early myogenic specification and determination is not affected in *Myog*-null embryos, but differentiation of the myoblasts and myofiber formation is abolished in vivo (Table 1) (Hasty et al., 1993; Nabeshima et al., 1993).

The first cells to express MRFs are located at the epaxial lip of the dermomyotome that activate *Myf5* when exposed to WNT signals from the neural tube and ectoderm, and SHH signals from the notochord (Fig. 1B) (Buckingham and Rigby, 2014; Ott et al., 1991; Tajbakhsh et al., 1998). The effector proteins of the WNT and SHH signaling pathways, GLI and TCF, respectively, recognize their binding sites on the early epaxial enhancer (EEE) of *Myf5* (-5.5 kb), activating its expression in these cells (Borello et al., 2006; Gustafsson et al., 2002). PAX3 also regulates *Myf5* expression epaxially via an indirect mechanism by which it activates the transcription of *Dmrt2* that in turn binds to the EEE of *Myf5* and positively regulates its expression (Sato et al., 2010). The cells committed to the myogenic lineage delaminate and migrate under the dermomytome initiating myotome formation. While *Myf5* expression in the epaxial domain of the dermomyotome can take place independently of PAX3, in the hypaxial domain, PAX3 binds directly to the -57.5 kb regulatory region of the *Myf5* gene required for appropriate MYF5 expression (Fig. 1B) (Bajard et al., 2006). The expression of PAX3 is essential not only for activating myogenic specification but also for cell survival (Relaix et al., 2004, 2005). Members of the Sine oculis homeobox (SIX) and the Eyes absent homologue (EYA) protein families, including SIX1, SIX4, EYA1 and EYA2 are co-expressed with PAX3 in the dermomyotome and regulate its expression (Fig. 1B) (Grifone et al., 2005, 2007). Overexpression of the SIX family member SIX1 activates *Pax3* in chick embryos (Heanue et al., 1999). In compound mouse mutants *Six1;Six4* and *Eya1;Eya2*, *Pax3* expression is lost and the embryos show a similar phenotype to *Pax3*-deficient embryos: a loss of the hypaxial lip domain and lack of limb muscles, showing that SIX and EYA transcription factors also lie genetically upstream of PAX3 (Grifone et al., 2007). SIX proteins also regulate myogenesis epaxially where SIX1 and SIX4 directly bind to regulatory sequences of MRF4 (Fig. 1B) (Grifone et al., 2005). In the *Six1;Six4* double knockout embryos, *Mrf4* expression is lost and consequently that of *Myod* is downregulated (Grifone et al., 2005; Relaix et al., 2013). In addition to the regulation of *Myod* expression by MRF4 epaxially; MRF4 and MYF5 can regulate MYOD expression hypaxially (Fig. 1B) (Kassar-Duchossoy et al., 2004).

In addition to the gene regulatory networks underlying skeletal muscle specification and commitment, recent studies on chromatin organization and architecture uncovered further players regulating myogenic gene expression. The ubiquitous expressed protein LIM domain binding protein 1 (LDB1) has been identified as a PAX3 interacting protein that is required for myogenic specification (Magli et al., 2019b). LDB1 regulates the formation of specific topologically associated domains (TAD) by mediating looping interactions at the *Pax3* locus and allowing PAX3 myogenic activity (Magli et al., 2019b). In addition, PAX3 and PAX7 are able to remodel chromatin accessibility in myogenic-associated loci in PSCs with induced PAX3 or PAX7 expression (Lilja et al., 2017; Magli et al., 2019a). In the case of PAX3, analysis of mesodermal cells derived from PAX3-overexpressing mouse ESCs showed that PAX3 increases chromatin accessibility in regions containing its binding sites and that this occurs in cooperation with SIX4 and TEA domain family member 2 (TEAD2) for robust myogenic commitment (Magli et al., 2019a). In mouse PSCs, overexpression of PAX7 leads to an increase in chromatin accessibility and acquisition of active histone modifications in muscle progenitors and committed myoblasts (Lilja et al., 2017). While some gene clusters, associated with myogenic determination, maintained these chromatin features if PAX7 activity was removed, other clusters required the permanent presence of PAX7 to sustain the histone modifications and accessibility (Lilja et al., 2017). Moreover, PAX7 binds to a majority of super-enhancers that contributes to the assembly of TADs associated with myogenic differentiation (Zhang et al., 2020). In addition to PAX3/7 proteins, MYOD can also regulate chromatin architecture. Regulatory regions of myogenic differentiation genes rely on MYOD interaction with the ubiquitous protein chromodomain helicase DNA binding 2 (CHD2) to modulate histone H3.3 variant incorporation into nucleosomes, which regulates downstream myogenic transcription (Harada et al., 2012). The incorporation of the histone variant H3.3 by the histone chaperone HIRA in regulatory regions of myogenic genes, such as *Pax7*, was further shown to be required to maintain myogenic cell identity (Esteves de Lima et al., 2021b). Furthermore, active chromatin marks on the regulatory regions of *Pax7* and Myf5 that rely on the methyltransferase MLL1, regulate their expression and satellite cell function in vivo (Addicks et al., 2019).

### Muscles of migratory origin

The migratory progenitors that contribute to the formation of muscles in distinct body regions are under distinct molecular regulation to maintain an undifferentiated state during migration and correctly reach the final location. This is the case of muscles like the diaphragm, the hypoglossal cord-derived tongue muscle and the limb muscles, with the former being the most studied and genetically understood. The skeletal muscles of the limbs are formed from the migration of progenitors that delaminate from the hypaxial dermomyotome of somites facing the limb buds, while the hypoglossal cord muscles derive from the occipital and cervical somites (Fig. 1A) (Chevallier et al., 1977; Christ et al., 1974; Relaix et al., 2003). In *Pax3*-deficient embryos migratory myogenesis is abolished (Bober et al., 1994; Relaix et al., 2003, 2004). Several gene regulatory networks operate downstream of PAX3 in the hypaxial dermomyotome to regulate muscle progenitor migration. PAX3 is phosphorylated by the serine-threonine kinase B-RAF and this is required for PAX3 gene target activation and progenitor migration (Shin et al., 2016). PAX3 controls the EMT of progenitor cells by directly regulating the expression of the tyrosine kinase receptor C-MET in the hypaxial progenitor cells required for migratory myogenesis (Bladt et al., 1995; Daston et al., 1996; Dietrich et al., 1999; Epstein et al., 1996; Yang et al., 1996). The controlled migration of the muscle progenitors towards the limb bud relies on the expression of the C-MET ligand, hepatocyte growth factor (HGF) in the limb bud mesoderm (Bladt et al., 1995; Brand-Saberi et al., 1996). In the *c-Met*- and in the *Hgf*-null embryos, delamination and migration of muscle progenitors do not occur, and migratory muscles are not formed, while axial muscles show no defects (Bladt et al., 1995; Dietrich et al., 1999). The homeodomain transcription factor ladybird 1 (LBX1) is expressed in the long-range migratory progenitors of the hypaxial dermomyotomes of the limbs, occipital and cervical somites and its expression is regulated by PAX3 (Jagla et al., 1995; Mennerich et al., 1998). In *Lbx1*-mutant embryos, migration of the muscles progenitors towards the limbs is impaired (Brohmann et al., 2000; Gross et al., 2000; Schäfer and Braun, 1999). In particular, progenitor cells that colonize the dorsal forelimb buds are absent, while ventrally, the progenitor cells are present but abnormally distributed, highlighting a cell-specific response to cues that guide migration and maintain the migratory potential of these cells (Brohmann et al., 2000). In addition, LBX1-regulated migration of the muscle progenitors towards the limb bud relies on LBX1 phosphorylation by FGF8 and the ERK pathway in chick embryos (Masselink et al., 2017). Another signaling pathway operating during muscle progenitor migration relies on the chemokine receptor CXCR4, whose expression is regulated by LBX1, and its ligand stromal cell-derived factor 1 (SDF1), expressed in the limb bud mesenchyme. CXCR4 is expressed in muscle progenitors that have already delaminated from the hypaxial dermomyotome and during migration towards the limb its expression co-localizes with LBX1 and PAX3 (Vasyutina et al., 2005).

### Embryonic and fetal myogenesis

Myogenesis occurs in successive and overlapping phases with two major waves of muscle formation, embryonic and fetal myogenesis. Embryonic skeletal muscle development starts at E10.5 in the mouse and relies on the PAX3-positive muscle progenitors (Biressi et al., 2007a). Upon differentiation, PAX3/PAX7-positive progenitors located in the myotome start to express MRFs, differentiate and fuse, giving rise to the first embryonic myofibers. Later, fetal myogenesis is initiated around E14.5 in the mouse, with muscle progenitors expressing PAX7 and differentiating into myoblasts that fuse between themselves and with the pre-existing embryonic fibers, allowing muscle growth (Biressi et al., 2007a). The presence of distinct progenitors that co-exist in the muscles but undergo differentiation at different developmental stages is regulated by intrinsic and extrinsic factors. The proliferation of PAX7-positive cells occurs preferentially at the muscle tips, which is specifically enriched in signals such as FGF and BMP (Edom-Vovard et al., 2001; Esteves de Lima et al., 2014, 2021c; Wang et al., 2010). During fetal myogenesis, PAX7-positive progenitor cells progressively exit cell cycle and by E16.5 they progressively adopt a satellite cell position, under the basal lamina (Esteves de Lima et al., 2014; Gros et al., 2005; Kassar-Duchossoy, 2005; Ontell and Kozeka, 1984; Picard and Marcelle, 2013; Relaix et al., 2005). This specific positioning of the progenitor cells under the basal lamina is regulated by the SIX1 and SIX4 transcription factors: in the *Six1;Six4*- null embryos PAX7-positive cells are impaired and do not adopt a homing position in the trunk muscles (Wurmser et al., 2020). In addition, NOTCH activity in progenitor cells is required to stimulate the production of their own basal lamina environment and to adhere to the fibers (Baghdadi et al., 2018; Bröhl et al., 2012).

The specific development of distinct muscle progenitor cell populations may be linked to differential response to external stimuli. For instance, exposing cultured myoblasts to ligands from the transforming growth factor (TGFβ) family (TGFβ1, BMP4) or to the phorbol ester TPA, leads to inhibition of fetal myoblast differentiation, while embryonic myoblasts are not affected (Biressi et al., 2007a). Genome-wide transcriptomic analysis performed in embryonic and fetal muscle progenitors isolated from mouse embryonic muscles identified groups of genes that were differentially expressed between these two populations (Biressi et al., 2007b). Embryonic myoblasts express high levels of the TGF and BMP inhibitors SMAD6 and SMAD7, which could explain the lack of effect of these molecules in culture (Biressi et al., 2007b). In addition, fetal myoblasts express high levels of PAX7 and genes associated with a more mature muscle phenotype including muscle creatine kinase (MKC), integrin α7 (ITGA7) and several laminins (Biressi et al., 2007b). Moreover, embryonic myoblasts that undergo differentiation express the slow myosin heavy chain (MYHC) isoform (MYH7) that is repressed in fetal myoblasts through a mechanism involving the fetal-specific transcription factor nuclear factor I X (NFIX) (Taglietti et al., 2016). NFIX is highly expressed in fetal but not embryonic myoblasts and it has been identified as a master regulator of the genetic switch between embryonic and fetal myogenesis (Biressi et al., 2007b; Messina et al., 2010; Taglietti et al., 2016). NFIX expression is directly activated by PAX7 binding to its promoter. Although PAX7 is sufficient to induce NFIX expression, NFIX is only mildly affected in *Pax7*-null embryos, showing that PAX7 is not required for NFIX expression, suggesting a possible overlapping activity (Messina et al., 2010). The small GTPase RHOA has been identified as a regulator of NFIX, by repressing the ERK signaling pathway, which positively regulates NFIX expression during fetal myogenesis (Taglietti et al., 2018).

### Major signaling pathways modulating myogenesis

During development, the balance between proliferation and differentiation of myogenic progenitors is tightly regulated to allow muscle growth while maintaining a pool of undifferentiated progenitors. Several signaling pathways play a role in the switch between proliferation and differentiation of skeletal muscle progenitor cells during development. The NOTCH signaling pathway is a major regulator of the muscle progenitor pool and inhibits myoblast differentiation in several model organisms (Bjornson et al., 2012; Bröhl et al., 2012; Delfini et al., 2000; Esteves de Lima et al., 2016; Hirsinger et al., 2001; Lahmann et al., 2019; Mourikis et al., 2012a, 2012b; Pascoal et al., 2013; Schuster-Gossler et al., 2007; Vasyutina et al., 2007). In the chick, overexpression of the NOTCH ligand DELTA1 inhibits myogenic differentiation in the somites and developing embryonic and fetal limb muscles (Delfini et al., 2000; Esteves de Lima et al., 2016; Hirsinger et al., 2001). Consistently, ablation of RBPJ, the NOTCH transcriptional co-effector, in muscle progenitors of mouse embryos leads to a premature shift towards differentiation and a progressive loss of the embryonic pool of PAX3/PAX7 progenitor cells (Vasyutina et al., 2007). Importantly, the number of muscle progenitors expressing MYOD is increased in these mutant embryos, and deleting *Myod* in the *Rbpj* conditional knockout rescues the phenotype and preserves the progenitor pool (Bröhl et al., 2012; Vasyutina et al., 2007). Moreover, in chick fetal development, signaling from NOTCH ligands expressed in the developing fibers are required to maintain the muscle progenitor cells in a NOTCH ON state and to avoid premature differentiation (Esteves de Lima et al., 2016). Conversely, expression of a constitutively active form of NOTCH (NOTCH intracellular domain, NICD) in myoblasts (MYF5-positve cells) leads to impaired muscle differentiation but maintains the progenitor pool (Mourikis et al., 2012b). In addition, mutating DELTA1 in mouse embryos leads to decreased muscle differentiation associated with the loss of progenitor cells (Schuster-Gossler et al., 2007). This is consistent with the concept that NOTCH signaling is very high in muscle progenitors and declines throughout differentiation (Mourikis et al., 2012a). The NOTCH downstream target hairy and enhancer of split-1 (HES1) is expressed in an oscillatory manner in developing and adult muscle stem cells (Lahmann et al., 2019). Cycling HES1 expression regulates the downstream target MYOD which consequently also oscillates; allowing the maintenance of the undifferentiated and proliferative state of the stem cells (Lahmann et al., 2019).

Other signaling pathways are also involved in maintaining muscle progenitors in a proliferative state, such as BMP signaling (Borok et al., 2020). During limb development, BMP signals from the ectoderm sustain the proliferation of PAX3-positive cells present in the muscle masses of chick embryos (Amthor et al., 1998). BMP gain- and loss-of-function experiments in chick fetal limbs demonstrated that increased BMP signaling leads to higher PAX7-positive cell number and increased muscle mass, while blocking BMP impairs muscle growth, suggesting a positive role of BMP in myogenic progenitor cell proliferation (Wang et al., 2010). In the mouse, BMP regulates post-natal growth and adult muscle homeostasis via regulation of satellite cell proliferation, while inhibiting BMP signaling impairs growth (Sartori et al., 2013; Stantzou et al., 2017). In addition, the BMP target gene inhibitor of DNA-binding protein 1 (ID1) is required to maintain satellite cells in a proliferative and undifferentiated state (Friedrichs et al., 2011; Ono et al., 2010).

The requirement for WNT signaling during myogenesis is distinct between embryonic and fetal phases (Hutcheson et al., 2009). Embryonic myoblasts in mouse developing limbs do not require WNT for correct progenitor and fiber formation, while fetal progenitor expression of a constitutively active form of β-catenin, an effector of the WNT pathway, increases PAX7-positive cell number (Hutcheson et al., 2009). In addition, WNT7a positively regulates the symmetric expansion of satellite cells during regeneration contributing to the maintenance of the stem cell pool (Le Grand et al., 2009). Muscle stem cell renewal is also regulated by SIX1 during muscle regeneration (Le Grand et al., 2012). More recently, post-translational modifications of PAX7 were also shown to regulate its activity and stem cell renewal. PAX7 acetylation by the acetyltransferase MYST1 regulates the satellite cell pool and the number of asymmetric cell divisions (Sincennes et al., 2021). Moreover, methylation of PAX7 by the arginine methyltransferase CARM1 is required for MLL1 recruitment to the *Myf5* locus to activate its expression following asymmetric cell division (Addicks et al., 2019; Kawabe et al., 2012).

### Recapitulating development *in vitro*

Research on muscle development has shifted to a regenerative medicine phase, where scientists modify the expression of genes essential for making muscle in vivo in order to recapitulate myogenesis in a petri dish. In vitro myogenesis protocols are now used both to grow functional muscles in the laboratory for tissue replacement, and to model muscle-associated diseases in vitro for cell therapy or drug discovery.

The conditional induction of PAX3 or PAX7 expression during mouse ESCs differentiation into embryoid bodies (EB), combined with cell sorting to enrich for paraxial mesoderm (PDGFRα-positive, FLK1-negative), efficiently produces a myogenic progenitor population (Table 2) (Darabi et al., 2011). Similarly, PAX7-conditional induction in mouse ESCs and PSCs robustly initiates the muscle program (Darabi et al., 2011; Kim et al., 2021; Lilja et al., 2017). Importantly, the muscle progenitors derived from PAX3 or PAX7-induced ESCs can contribute to muscle regeneration when grafted into the muscles of the Duchenne dystrophy mouse model (*mdx*) that lacks the dystrophin protein. When engrafted, these cells are capable of generating healthy fibers that express dystrophin and replenish the satellite cells which once again adopt a position under the basal lamina (Darabi et al., 2008, 2011; Filareto et al., 2013; Kim et al., 2021). These are important findings for using these techniques for regenerative medicine and ex vivo gene therapy approaches.

**Table 2:**
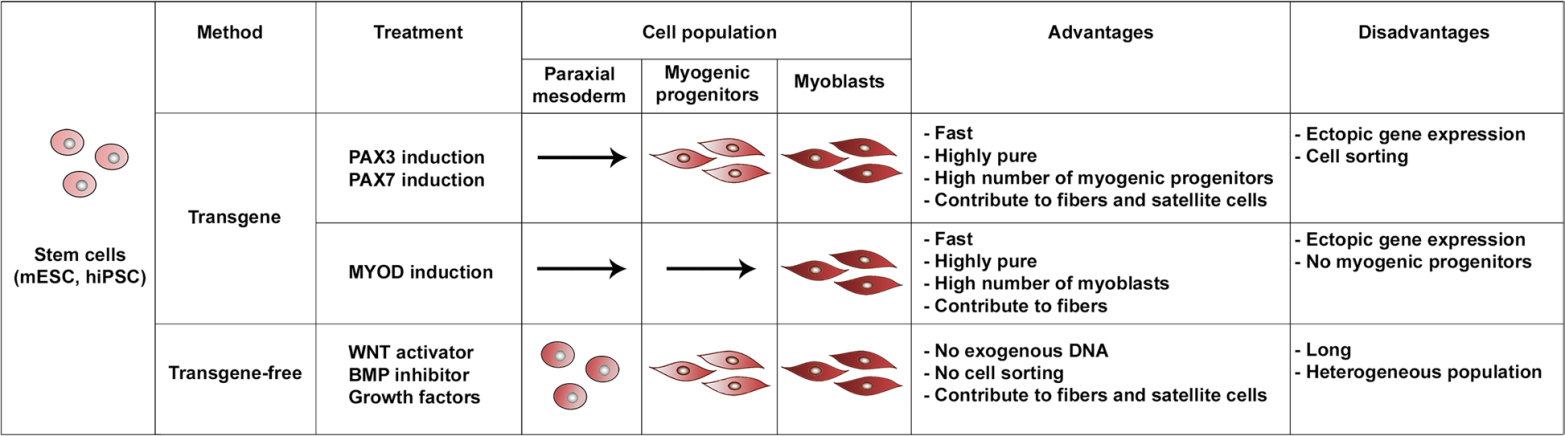
Overview of the different established protocols to differentiate myoblasts from pluripotent stem cells. Arrows represent the bypass of the associated cell population on each protocol

Ectopic expression of MYOD is also able to drive human induced PSCs or mouse ESCs into myogenic differentiation (Table 2) (Albini et al., 2013; Dekel et al., 1992; Goudenege et al., 2012; Tanaka et al., 2013; Warren et al., 2010). This method allows one to bypass the EB and mesodermal differentiation steps required in other protocols, leading to direct and efficient generation of myoblasts. The ectopic expression of BAF60C, a chromatin remodeler from the SWI/SNF family and MYOD cofactor, in induced PSCs allows chromatin remodelling at target myogenic genes and activation of their expression (Albini et al., 2013). However, while MYOD-expressing PSCs are able to engraft and give rise to muscle fibers, the capacity of MYOD-positive cells to contribute to the stem cell pool has not been addressed (Goudenege et al., 2012). Concerns regarding the use of transgene methods in disease therapy are associated with the innate response to viral particles when using viruses to deliver the genetic material and the risk of genomic recombination when delivering DNA. In order to bypass innate anti-viral responses, the use of synthetic and modified *MYOD1* messenger RNA combined with interferon inhibitors efficiently directed hiPSC (human induced pluripotent stem cells) differentiation into myoblasts (Warren et al., 2010). To overcome concerns associated with random genomic integration of transgenes, a promising strategy uses specific targeting to genomic safe harbors sites. The targeting of *PAX7* to such genomic regions provided myoblasts displaying high efficiency in vivo engraftment, an alternative approach to generate PSC-derived myogenic progenitors that could potentially be of therapeutic use (Kim et al., 2021).

Differentiation of embryonic stem cells or induced pluripotent stem cells into the myogenic lineage without the use of transgenes was defined in distinct protocols (Table 2) (Caron et al., 2016; Chal et al., 2016, 2018; Hicks et al., 2018; Shelton et al., 2014; Zhao et al., 2020). The knowledge generated from developmental studies allowed the design of protocols that attempt to recapitulate myogenesis by treating pluripotent stem cells with specific molecules at precise time-points. To initiate presomitic mesoderm commitment, epiblast-like cells are treated with the WNT activator (CHIR99021) and the BMP inhibitor (LDN193189) (Chal et al., 2016). Following addition of a cocktail of growth factors to the cultures (FGF2, HGF and IGF1), the cell population presents a mix of progenitor cells (PAX7-positive) and differentiated myocytes (MYOG-positive). Transgene-free protocols have been shown to be effective in generating myoblasts capable of restoring Dystrophin in fibers of DMD-deficient mice by grafting PAX7 + cells (Pax7-GFP reporter) and MYF5 + cells (Myf5-tdTomato reporter line) from late stage differentiation cultures (Chal et al., 2015; Zhao et al., 2020).

Although the use of transgene-free cultures to differentiate stem cells into the myogenic lineage has advantages regarding a putative future use for cell therapy, the significant degree of contamination by other cell types may require cell sorting to isolate pure myogenic populations. By using a *MYF5*-hiPSC reporter line, a recent single cell RNA-sequencing (scRNA-seq) study identified the surface markers FGFR4 and CDH13 as being expressed in myogenic progenitors. Furthermore, cells purified with each one of these markers were able to generate Dystrophin-positive fibers when transplanted into an immunodeficient mouse model for DMD (Nalbandian et al., 2021).

One common drawback of most iPSC-differentiation protocols is the inability to produce mature cell types in vitro. This is also the case for myogenic differentiation protocols that do not yield fully differentiated myotubes in vitro and prevents a deeper analysis of the late myogenic differentiation phases. In fact, the myotubes present in these cultures usually have an embryonic or early fetal phenotype and lack the capacity to mature into late fetal or adult myofibers. Transcriptomic analysis of human PSC-derived myogenic progenitors and human fetal myoblasts highlighted differences in the expression of signaling pathways components between these two populations (Hicks et al., 2018). Human fetal myoblasts downregulate the expression of TGFβ signaling activators while human PSC-derived myogenic progenitors express high levels of these growth factors. When culturing human PSC-derived myogenic progenitors with a TGFβ inhibitor, the myotubes obtained are thicker, have increased levels of expression of fetal and adult MYHC and present a higher level of sarcomeric organization (Hicks et al., 2018). Moreover, the combination of prednisolone, with the aim of recapitulating the glucocorticoid signaling burst operating during human fetal development, and TGFβ inhibitor in the differentiation culture medium significantly improved the morphology of hiPSC-derived fibers towards a more mature phenotype (Al Tanoury et al., 2021). Gene expression profiling from hiPSC-derived MYF5 + cells (Myf5-tdTomato reporter line) revealed that late-stage cultured MYF5 + cells presented a fetal myogenic stem cell phenotype and engrafted with higher efficiency than early-stage cultured MYF5 + cells (Zhao et al., 2020). In addition to the transcriptomic differences, the inability to achieve mature myotubes in vitro can also be associated with the lack of positional information cues that operate in vivo to regulate muscle specification and the metabolic complexity of adult muscle fibers. In fact, single-nuclei RNA-seq analysis of adult muscle fibers revealed the presence of distinct myonuclear domains, highlighting the complexity of the transcriptome of the myonuclei along the fiber (Cramer et al., 2020; Dos Santos et al., 2020; Kim et al., 2020). The question of whether the regionalization of the nuclei within the myotube can be modulated in vitro remains a question and a challenge for the field.

The optimization of protocols to differentiate human PSCs into myoblasts for use in therapeutic approaches also relies on the understanding of the molecular networks operating during human myogenesis that may differ from those of the mouse. Therefore, the development of databases and resources to increase the knowledge of human myogenesis is of major importance. Recently, a scRNA-seq experiment performed in embryonic, fetal and postnatal human skeletal muscles allowed the mapping of myogenic differentiation trajectories of human myoblasts (Xi et al., 2020). These data can be compared to those of the PSC-derived myoblast populations generated in vitro and will contribute to the optimization of protocols that aim to produce myogenic cells from PSCs (Xi et al., 2020).

## Conclusions

The development of skeletal muscle progenitors is tightly regulated and relies on the hierarchical expression of genes that coordinate their specification and commitment. The role of the PAX genes as well as that of the MRF transcription factors regulating myogenesis is well established. In addition, several genome-wide analyses for PAX3 and PAX7 binding sites have been performed, revealing many potential PAX3/PAX7 target genes. However, only recently has the muscle field started to appreciate the role of these factors as tissue-specific chromatin remodeling factors that rely on interactions with ubiquitous chromatin architecture regulators. Furthermore, the identification of upstream regulators of PAX3 and PAX7 as well as their regulatory regions remains to be done. The answers to these questions could lead to the understanding of how these genes act and regulate downstream targets by inter-playing with chromatin remodelers and also how they are regulated in different developmental contexts.

## Data Availability

Not applicable.

## Abbreviations

EMT: Epithelial-to-mesenchymal transition
PSCs: Pluripotent stem cells
MRF: Myogenic regulatory factors
bHLH: Basic helix-loop-helix
EEE: Early epaxial enhancer
ESCs: Embryonic stem cells
ChIP-seq: Chromatin immunoprecipitation-sequencing
TAD: Topologically associated domains
MYHC: Myosin heavy chain
EB: Embryoid body
hiPSCs: Human induced pluripotent stem cells
scRNA-seq: Single cell RNA-sequencing
